# Risk Prediction of Myelosuppression Following First-line Chemotherapy in Colorectal Cancer

**DOI:** 10.7150/jca.104412

**Published:** 2025-01-20

**Authors:** Yanyuan Du, Yuming Liu, Ruiying Fang, Liu Cai, Ying Song, Susu Ma, Huibo Yu, Jin Gao, Hongtai Xiong, Hanyue Zhang, Baihui Li, Honggang Zheng

**Affiliations:** 1 Department of Oncology, Guang'anmen Hospital, China Academy of Chinese Medical Sciences, Beijing 100053, China.; 2 Beijing University of Chinese Medicine, Beijing 100029, China.

**Keywords:** Colorectal cancer (CRC), Chemotherapy-induced myelosuppression, Predictive nomogram, Risk factors, Retrospective study

## Abstract

**Background:** Colorectal cancer (CRC) is a leading cause of cancer-related deaths, with over 1.9 million new cases and 904,000 deaths in 2022. Chemotherapy is a primary treatment for CRC but often leads to myelosuppression, significantly affecting treatment efficacy and patient outcomes. Predictive tools for chemotherapy-induced myelosuppression are currently lacking.

**Methods:** This retrospective study analyzed 855 CRC patients from Guang'anmen Hospital who received first-line chemotherapy (CapeOx, FOLFOX, FOLFIRI) between April 2020 and July 2024. Patients were divided into training (684) and validation (171) groups. Univariate analysis, LASSO regression, and multivariable logistic regression identified risk factors for myelosuppression, and a predictive nomogram was developed and validated using ROC curves, calibration curves, and decision curve analysis. Propensity score matching (PSM) was employed to minimize baseline differences between groups, followed by multivariate logistic regression analysis on the post-PSM data.

**Results:** The incidence of myelosuppression was similar in both groups (33.04% vs. 32.16%). Significant predictors included age, smoking, diabetes, BMI, tumor location, lung metastasis, albumin (ALB) levels, and carcinoembryonic antigen (CEA) levels. The nomogram demonstrated good predictive performance with AUC values of 0.78 and 0.80 for the training and validation groups, respectively, showing consistent and clinically useful predictions. PSM further validated the robustness of the model, confirming BMI as a consistently significant predictor of myelosuppression.

**Conclusions:** The study identified key risk factors for chemotherapy-induced myelosuppression in CRC patients and developed a nomogram for prediction. This tool can help clinicians assess risk and guide treatment decisions. Limitations include potential selection bias and the need for external validation in diverse populations. Future studies should further refine and validate this predictive model.

## Introduction

Colorectal cancer (CRC) refers to malignant tumors occurring in the colon or rectum. It ranks third in incidence, following lung cancer and breast cancer, and is the second leading cause of cancer-related deaths. Statistics indicate that in 2022, there were over 1.9 million new cases of colorectal cancer (including anal cancer) and 904,000 deaths, accounting for nearly one-tenth of all cancer cases and deaths^1^. With the aging population and changes in lifestyle, the incidence and mortality rates of CRC are expected to increase significantly in the coming decades. By 2040, the global incidence of CRC is projected to rise to 3.2 million new cases, with 1.6 million deaths^2^-^3^. Evidence suggests that in many high-income countries, the incidence of CRC among individuals under 50 years old has been increasing annually^4^-^5^. CRC has thus become an increasingly serious public health issue.

Chemotherapy is one of the main treatment options for CRC. For patients with early-stage CRC (Stage I and Stage II), surgical resection is the primary treatment modality. However, for high-risk Stage II patients, adjuvant chemotherapy post-surgery may be recommended to reduce the risk of recurrence. For patients with locally advanced CRC (Stage III), the standard treatment regimen includes surgical resection combined with adjuvant chemotherapy. In cases of metastatic CRC (Stage IV), systemic chemotherapy is the preferred approach, often in combination with targeted therapies such as bevacizumab or cetuximab^6^-^7^. The first-line treatment regimens for CRC include FOLFOX (fluorouracil [5-FU], leucovorin, and oxaliplatin), FOLFIRI (fluorouracil [5-FU], leucovorin, and irinotecan), and CapeOx (capecitabine and oxaliplatin)^6^. Although the aforementioned regimens demonstrate significant clinical efficacy^8^-^9^, they are still associated with side effects such as myelosuppression, gastrointestinal reactions, dermatological lesions, and peripheral neurotoxicity^10^-^12^. In some cases, these regimens can lead to life-threatening cardiotoxicity^13^ and central nervous system toxicity^14^. Among the side effects, myelosuppression is one of the most common adverse reactions following first-line chemotherapy regimens for CRC. Myelosuppression is a side effect induced by chemotherapy or radiotherapy that results in a reduced ability of the bone marrow to produce blood cells, leading to pancytopenia. The primary cause of myelosuppression is that chemotherapy, while attacking tumor cells, also inhibits the highly proliferative and poorly differentiated bone marrow cells, suppressing all immature cells with proliferative capabilities^15^. Specific manifestations of myelosuppression include neutropenia, anemia, and thrombocytopenia^16^, which can also cause adverse reactions such as fever, rash, and bone pain^17^-^18^. More severely, the reduction of neutrophils and platelets can significantly increase the risk of infection and bleeding, directly threatening the patient's life. Furthermore, when severe myelosuppression occurs, it necessitates a reduction in chemotherapy dosage or a delay in treatment, impacting the clinical benefits of the therapy. Therefore, early diagnosis of chemotherapy-induced myelosuppression is of paramount importance.

**Figure 1:**
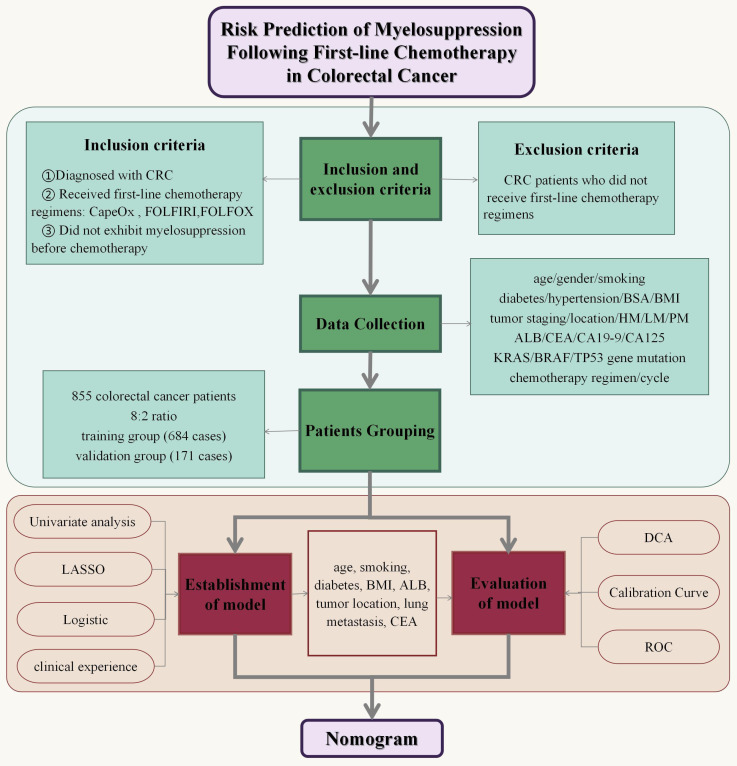
Flowchart of the study protocol.

Considering the crucial role of chemotherapy in treating CRC patients and the adverse effects of chemotherapy-induced myelosuppression, it is imperative to identify predictive factors for myelosuppression in this population. Currently, there are no reliable methods to accurately assess the risk of myelosuppression in CRC patients. Creating a reliable predictive tool for assessing myelosuppression risk after chemotherapy would be of significant clinical importance. This study aims to develop and validate a model for predicting the myelosuppression after first-line chemotherapy for colorectal cancer. The study protocol of this article is shown in Figure 1.

## Methods

### Patients and study design

This study retrospectively selected 855 inpatients from the Oncology Department of Guang'anmen Hospital, China Academy of Chinese Medical Sciences, from April 2020 to July 2024. To maximize the accuracy, sensitivity, and specificity of the predictive model and enhance its overall performance, including its utility in clinical decision-making, this study utilized the randomizr package in R to perform simple randomization of the included cases (training group: validation group = 8:2). This approach reduces selection bias, improves comparability between groups, and enhances the predictive power of the model's features with respect to the outcome indicators, thereby increasing the external validity of the nomogram predictive model. The patients were randomly divided into a training group and a validation group at a ratio of 8:2. Inclusion criteria: ① Diagnosed with CRC according to the diagnostic criteria of the "Chinese Protocol of Diagnosis and Treatment of Colorectal Cancer (2023 Edition)"; ② Received first-line chemotherapy regimens: CapeOx (capecitabine and oxaliplatin), FOLFOX (fluorouracil, leucovorin, and oxaliplatin), FOLFIRI (fluorouracil, leucovorin, and irinotecan); ③ Did not exhibit myelosuppression before chemotherapy, defined as meeting all of the following criteria: adult peripheral blood leukocyte count ≥4.0×10^9/L, absolute neutrophil count ≥2.0×10^9/L, platelet count ≥100×10^9/L, hemoglobin ≥115g/L (female) or hemoglobin ≥130g/L (male). Exclusion criteria: CRC patients who did not receive first-line chemotherapy regimens.

The training group consisted of 684 patients and was used to develop and train the model. The validation group consisted of 171 patients and was used to validate the model.

The study was approved by the ethical committee of our hospital (Approval Number: 2022-215-KY).

### Outcome definition

The outcome variable of this study is antineoplastic drug-related myelosuppression, defined as adult peripheral blood leukocyte count <4.0×10^9/L, absolute neutrophil count <2.0×10^9/L, platelet count <100×10^9/L, and hemoglobin <115g/L (female) or hemoglobin <130g/L (male). Meeting any one of these four criteria qualifies as a diagnosis of antineoplastic drug-related myelosuppression^16^. Laboratory tests were evaluated between the start of each chemotherapy cycle and the next cycle, typically conducted 3-7 days after chemotherapy, to assess the severity of myelosuppression following first-line chemotherapy.

### Demographic and clinical measures for prediction and defnition

The predictive variables include patient-specific and chemotherapy regimen-related risk factors. The candidate predictors include age, gender, smoking, diabetes, hypertension, body surface area (BSA), body mass index (BMI), tumor staging, tumor location, hepatic metastasis, lung metastasis, peritoneal metastasis, albumin (ALB), carcinoembryonic antigen (CEA), carbohydrate antigen 19-9 (CA19-9), carbohydrate antigen 125 (CA125), KRAS gene mutation, BRAF gene mutation, TP53 gene mutation, chemotherapy regimen, and chemotherapy cycle. These predictive variables were assessed and recorded before each chemotherapy session.

Age was categorized into three groups: <60 years, 60-74 years, and ≥75 years. Smoking status included both past and current smoking habits. BSA was divided into three categories: <1.6 m², 1.6-1.8 m², and >1.8 m². According to the Chinese standard^19^, BMI was categorized into non-overweight and overweight, with a cutoff point of 24.0 kg/m². CRC staging was based on the AJCC Cancer Staging Manual, 8th Edition^20^, and divided into four stages: Stage I, II, III, and IV. Tumor location was categorized into five anatomical sites: ascending colon, transverse colon, descending colon, sigmoid colon, and rectum. The first-line chemotherapy regimens for CRC were categorized into three groups: CapeOx (capecitabine and oxaliplatin), FOLFOX (fluorouracil, leucovorin, and oxaliplatin), and FOLFIRI (fluorouracil, leucovorin, and irinotecan). The number of chemotherapy cycles was divided into three categories: 1-2 cycles, 3-4 cycles, and 5 or more cycles.

### Statistical analysis

Univariate analysis was conducted to preliminarily identify potential risk factors for myelosuppression following first-line chemotherapy in CRC patients. Subsequently, to identify the most predictive factors associated with myelosuppression in CRC patients undergoing first-line chemotherapy, we applied the Least Absolute Shrinkage and Selection Operator (LASSO) regression model. This statistical approach is particularly effective in selecting relevant non-zero features, thereby enhancing the model's predictive accuracy by focusing on the most significant variables contributing to myelosuppression risk in this patient population^21^-^22^. Based on the univariate analysis and the non-zero features identified, combined with clinical experience, a multivariable logistic regression model was established for further analysis. In the multivariable logistic regression analysis, the relationship between each factor and the outcome variable was determined using forward selection, backward elimination, and stepwise selection methods. The likelihood ratio test with the minimum Akaike Information Criterion (AIC) value was used as the stopping rule^23^. A nomogram model was then constructed, incorporating the results from both the LASSO regression analysis and the multivariable logistic regression analysis.

To assess the discriminatory capability of the developed nomogram, we analyzed the area under the receiver operating characteristic (ROC) curve. This metric provides a quantitative measure of the nomogram's ability to distinguish between different clinical outcomes, offering a robust evaluation of its predictive accuracy and effectiveness in clinical practice. Calibration was assessed by comparing observed outcomes with predicted probabilities, with an optimal fit indicated by an intercept of α=0 and a slope of β=1. To thoroughly evaluate the clinical utility and applicability of the nomogram, we employed decision curve analysis (DCA). This analytical method offers a nuanced approach to assessing the net benefit by quantifying it across a comprehensive spectrum of threshold probabilities present within the dataset. By doing so, DCA provides a more detailed and practical understanding of the nomogram's performance in various clinical scenarios, ensuring that its predictive accuracy and potential benefits are effectively validated in real-world settings. This approach underscores the robustness and reliability of the nomogram in guiding clinical decision-making^24^-^25^.Due to certain differences in specific factors between patients with myelosuppression and those without, we employed a propensity score matching (PSM) analysis model using a caliper of 0.2 to balance the intergroup variability of these factors. This approach was aimed at eliminating potential selection bias and enhancing the evidence level of our retrospective study. Matching was performed using the "nearest-neighbor" method, with a PSM ratio of 1:1. Subsequently, a multivariable logistic regression analysis was conducted on the post-PSM baseline data to further validate the original logistic regression model and the nomogram.

The organization of data and execution of statistical analyses were conducted using Zstats software alongside R version 4.4.0. For the univariate analysis, continuous variables were assessed using the Wilcoxon rank-sum test, while categorical variables were analyzed with the χ² test. In the multivariable logistic regression analysis, we employed forward selection, backward elimination, and stepwise selection techniques, determining the optimal model based on the minimum AIC value. The calibration curve was assessed using the unreliable U test, ensuring the model's calibration accuracy. The "rms" package in R facilitated the creation of the nomogram and calibration curve. A *p*-value of less than 0.05 was considered statistically significant throughout the analysis.

## Results

### Characteristics of patients

In this study, a retrospective selection of 684 patients was made for inclusion in the training group, while 171 patients were assigned to the validation group. There were no statistically significant differences in demographic and clinical characteristics between the two groups (*P*>0.05). Additionally, the incidence of myelosuppression did not differ significantly between the training and validation groups (33.04% vs. 32.16%, *P*=0.827) (Table 1).

### Feature selection

In the univariate analysis of the training group of CRC patients after first-line chemotherapy, significant differences were observed between the myelosuppression and non-myelosuppression groups in terms of age, gender, smoking, diabetes, body surface area(BSA), body mass index(BMI), tumor staging, tumor location, hepatic metastasis, lung metastasis, albumin(ALB), carcinoembryonic antigen(CEA), and the number of chemotherapy cycles. No statistically significant differences were found between the two groups in terms of hypertension, peritoneal metastasis, carbohydrate antigen 19-9(CA19-9), carbohydrate antigen 125(CA125), KRAS gene mutation, BRAF gene mutation, TP53 gene mutation, or chemotherapy regimen (Table 2).

**Figure 2:**
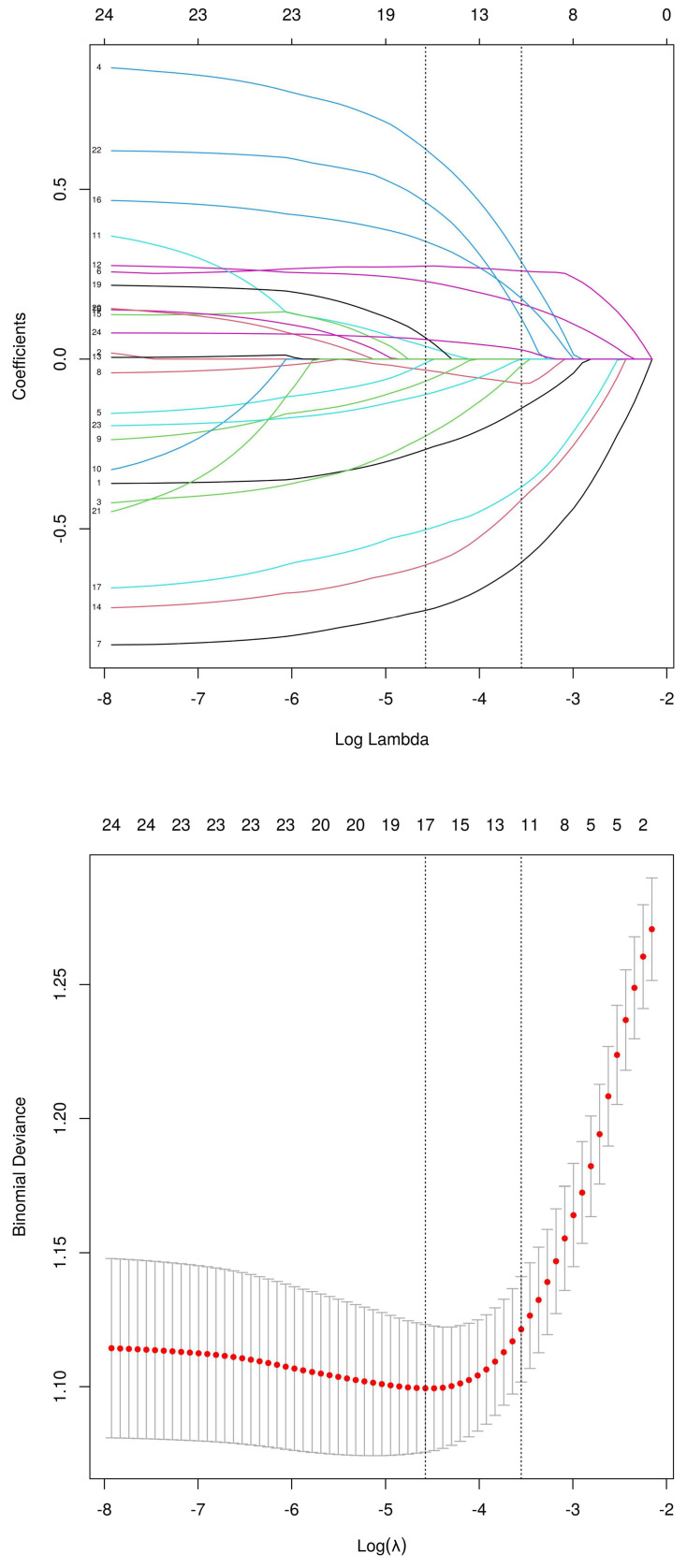
Demographic and Clinical Characteristics Screening Using the LASSO Regression Model. (A) LASSO regression coefficient path diagram of risk factors. (B) Cross-validation curve for the LASSO regression.

Using the LASSO regression model, the 24 sociodemographic and clinical characteristics were reduced to 12 potential risk factors with non-zero coefficients (approximately 2:1 ratio) (Figure 2). These features include age, smoking, diabetes, BSA, BMI, tumor staging, tumor location, lung metastasis, ALB, CEA, TP53 gene mutation, and the number of chemotherapy cycles.

### Risk factors for myelosuppression following first-line chemotherapy in CRC patients

In the multivariate analysis, this study employed a multivariate logistic regression analysis method, using myelosuppression as the dependent variable. The independent variables included age, smoking, diabetes, BSA, BMI, tumor staging, tumor location, hepatic metastasis, lung metastasis, ALB, CEA, and the number of chemotherapy cycles. The results indicated that age, smoking, diabetes, BMI, tumor location, lung metastasis, ALB, and CEA were independent risk factors for myelosuppression in CRC patients undergoing first-line chemotherapy (Table 3).

### Establishment of the nomogram for predicting myelosuppression after first-line chemotherapy in CRC patients

By synthesizing the outcomes of univariate analysis, LASSO regression, and multivariate logistic regression, and incorporating clinical expertise, the study identified age, smoking, diabetes, BMI, tumor location, lung metastasis, ALB, and CEA as independent risk factors for myelosuppression following first-line chemotherapy in CRC patients. These eight risk factors were incorporated into a predictive model, culminating in the creation of a nomogram for forecasting myelosuppression in CRC patients after first-line chemotherapy (Figure 2).

The model uses the 8 risk factors as variable axes. Each variable's score is vertically aligned with the score axis, which ranges from 0 to 100 points. The total score of the eight variables corresponds to the total score axis, ranging from 0 to 500 points. The total score is then vertically aligned with the probability axis, which ranges from 0.1 to 0.9, indicating the probability of myelosuppression. The total score of each variable corresponds to the risk probability line to obtain the probability of risk of myelosuppression. For example, for a 90-year-old CRC patient with lung metastasis, a BMI of 25 kg/m², a history of type 2 diabetes, chronic smoking, and an albumin level (ALB) of 32 g/L, the nomogram scores are as follows: age 90 years (47.5 points), lung metastasis (32 points), BMI 25 kg/m² (54 points), type 2 diabetes (27.5 points), smoking (27 points), and ALB 32 g/L (22 points), totaling 210 points, resulting in a 0.13 (13%) probability of myelosuppression. Thus, both doctors and patients can use this intuitive and user-friendly scoring system to predict the risk of myelosuppression and rationalize clinical decisions regarding any adjunctive treatment before or during chemotherapy. Therefore, this nomogram has clinical value for predicting and preventing myelosuppression in CRC patients after first-line chemotherapy.

### Evaluation of the nomogram for predicting myelosuppression after first-line chemotherapy in CRC patients in the training and validation groups

**Figure 4:**
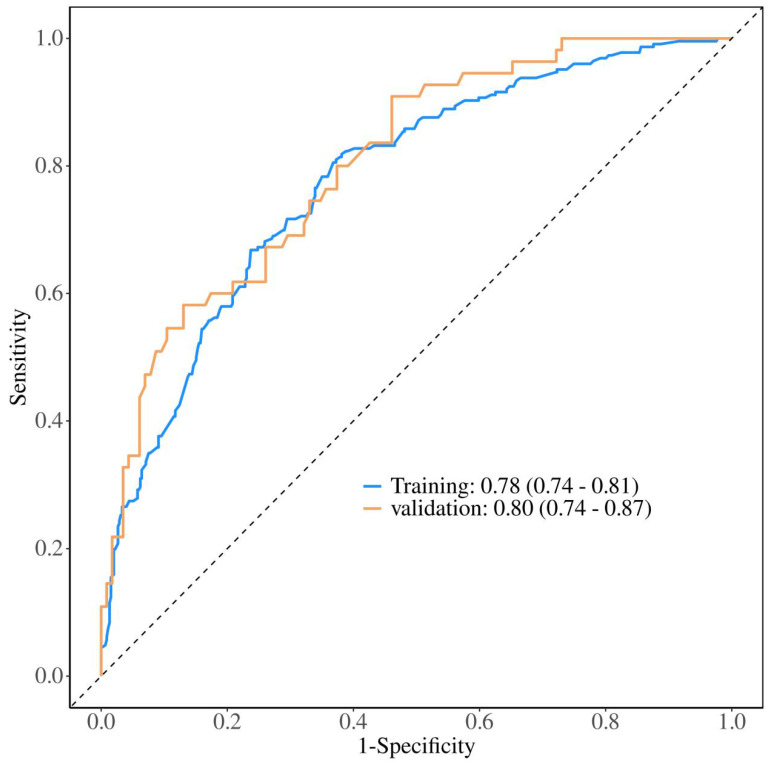
ROC curves for the training and validation groups. ROC curve for the training group, AUC = 0.78; ROC curve for the validation group, AUC = 0.80.

**Figure 5:**
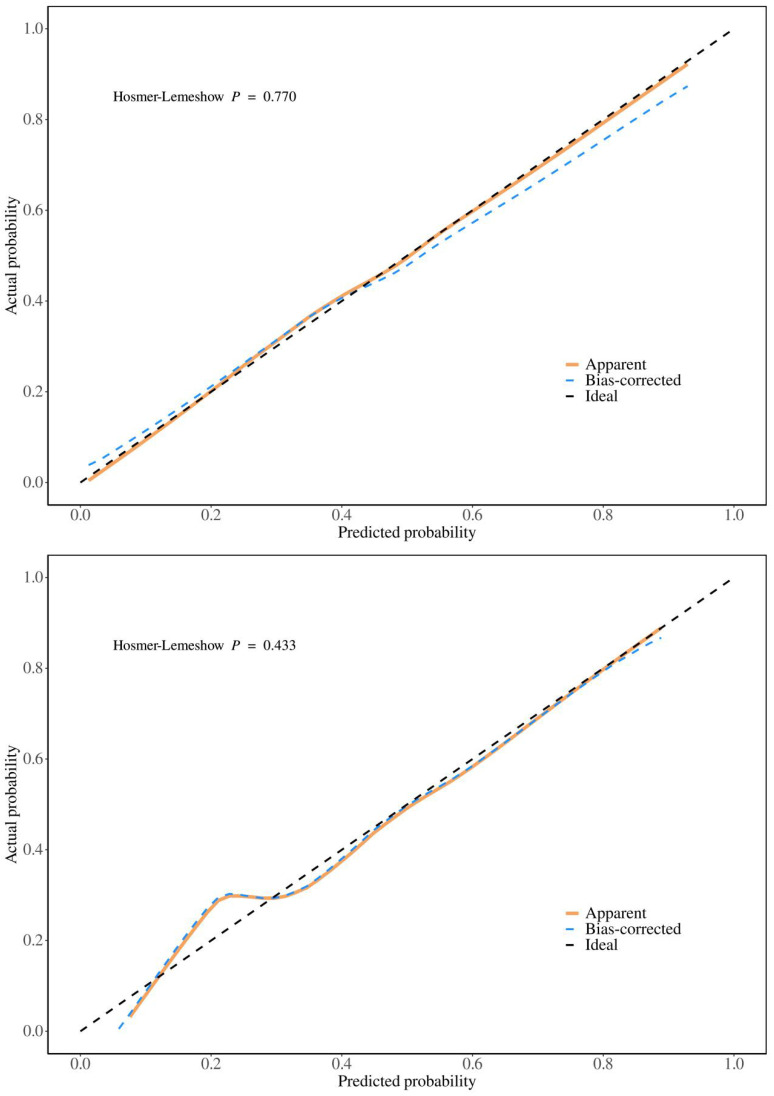
Calibration curves for training and validation groups. (A) Calibration curve for the training group (B) Calibration curve for the validation group.

**Figure 6:**
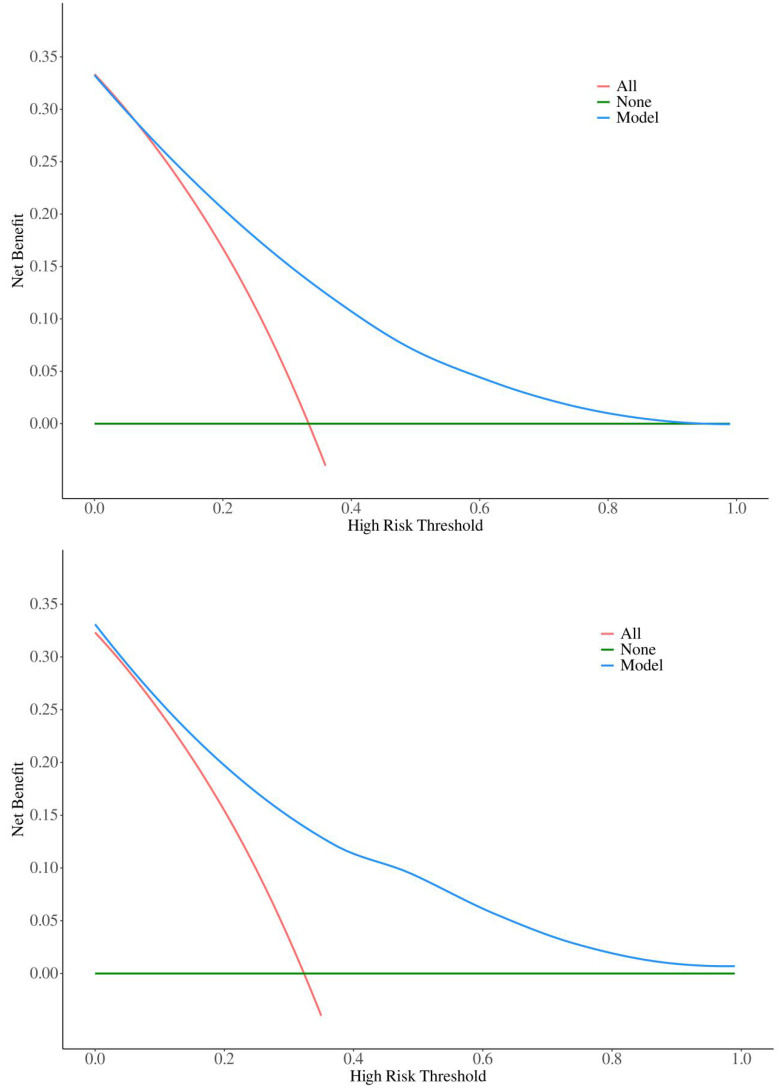
Decision curves for the training and validation groups. (A) Decision curve for the training group (B) Decision curve for the validation group.

To validate the sensitivity and specificity of the nomogram model, this study evaluated the predictive nomogram using the area under the ROC curve (AUC). The AUC values of the nomogram for the training and validation groups were found to be 0.78 and 0.80, respectively (Figure 5). The calibration curves demonstrated acceptable consistency between the predicted and actual values in two groups of CRC patients receiving first-line chemotherapy (Figure 4). DCA showed that if the threshold probability (Pt) in the training and validation groups ranged between 10-90%, timely clinical intervention based on the predicted probability from the nomogram before first-line chemotherapy could provide a higher net benefit compared to either the full intervention or no intervention strategies (Figure 6). Therefore, the nomogram model exhibits good discriminative ability, acceptable calibration, and clinical utility in predicting myelosuppression in CRC patients following chemotherapy.

### Risk factors for myelosuppression after first-line chemotherapy in CRC patients following PSM

After PSM, all variables achieved balance between the groups, with P-values greater than 0.05 and standardized mean differences (SMDs) generally below 0.10 (Table 4).

We conducted a multivariate logistic regression analysis on data after PSM and reconfirmed that BMI is a significant risk factor for bone marrow suppression in CRC patients following first-line chemotherapy. It is important to emphasize that BMI consistently emerged as a significant risk factor in both the initial model and the model optimized for baseline variables, indicating its critical role in the occurrence of chemotherapy-induced bone marrow suppression.

## Discussion

Despite recent advancements in treatment options, CRC continues to significantly impact public health due to its high incidence and mortality rates. Currently, standard treatment regimens rely on chemotherapy protocols containing highly myelotoxic drugs, such as FOLFOX and FOLFIRI. These treatments often lead to myelosuppression, particularly in elderly patients with multiple comorbidities. Therefore, identifying risk factors for myelosuppression in CRC patients following first-line chemotherapy is crucial for optimizing treatment outcomes and improving survival rates.

This study aimed to identify risk factors for chemotherapy-induced myelosuppression in CRC patients. Through univariate and multivariate logistic regression analyses, we identified eight significant risk factors: age, smoking status, diabetes, BMI, tumor location, lung metastasis, ALB, and CEA. Nomograms, which graphically represent the relationships between multiple variables using a statistical model, are widely used in clinical practice due to their user-friendly interface, high accuracy, ease of prognostic interpretation, and utility in guiding clinical decision-making. This study seeks to develop and validate such a nomogram, aiming to predict the probability of myelosuppression and aid in individualized treatment decisions. The predictive model incorporates eight easily obtainable demographic and clinical variables, enabling accurate and personalized risk assessment for myelosuppression post-chemotherapy in CRC patients. Meanwhile, the large sample size of our group enhances the nomogram's applicability and accuracy. This study also utilized AUC, calibration curves, and DCA to validate the model. The AUC is a widely used metric for evaluating the performance of predictive models, providing a single value that quantifies the model's ability to distinguish between positive and negative cases^26^. The AUC of the training group in this model is 0.78, indicating good generalization without overfitting, while the AUC of the validation group is 0.80, confirming strong performance on new data. The small difference between the training and validation AUCs suggests that the model is robust. Calibration curves assess how well predicted probabilities align with actual outcomes. A well-calibrated model produces predictions that match observed results, ideally falling on a diagonal line representing perfect calibration^27^. In this model, both the training and validation groups show calibration curves that closely follow the ideal line, indicating good calibration. The p-values for the training and validation groups (0.770 and 0.433, respectively) further support that there is no significant deviation from good calibration. DCA evaluates the clinical utility of the model by comparing its net benefit across different risk thresholds to two extreme strategies: treating all patients or treating none^28^. The model provides a higher net benefit within the threshold probability range of 10-90%, indicating that it supports more effective decision-making, helps to personalize interventions, reduces unnecessary treatments, and balances the benefits and risks of clinical interventions.

Finally, to minimize the impact of baseline imbalance on the results of multivariate analysis, we employed PSM to balance baseline characteristics. In the matched dataset with balanced baselines, we conducted a multivariate logistic regression analysis again, and the results indicated that BMI remained a significant risk factor. Although several other significant factors were identified in the initial model, this does not imply that they were false associations due to confounding bias. On the contrary, these factors may indeed have an influence on the occurrence of myelosuppression in specific subgroups of patients, but their significance was weakened in the dataset with balanced baseline characteristics. The persistent significance of BMI in the optimized model reflects its consistency and independence under varying baseline conditions.

Age is one of the risk factors for chemotherapy-induced myelosuppression identified in this study. Chemotherapy-related myelosuppression in elderly patients may be associated with decreased metabolism of myelosuppressive drugs, leading to drug accumulation and increased toxicity^29^. Decline in bone marrow function and depletion of hematopoietic stem cell reserves are also reasons for increased susceptibility to myelosuppression in the elderly. This decline results from changes in the bone marrow microenvironment and alterations in the intrinsic properties of hematopoietic stem cells. Chronic low-grade inflammation and elevated levels of oxidative stress commonly present in the elderly^30^, as well as diminished DNA damage repair capacity^31^, can impair the bone marrow microenvironment and hematopoietic stem cell function, making myelosuppression more likely. Furthermore, elderly patients often have multiple chronic diseases, and medications used to treat these conditions, such as methotrexate and allopurinol, may suppress bone marrow function or exacerbate myelosuppression through drug interactions^32^.

The impact of smoking on chemotherapy-induced myelosuppression is also noteworthy. While smoking is generally associated with altered peripheral blood cell counts, such as increased neutrophils and erythrocytes^33^, this does not imply a protective effect of smoking on bone marrow function. On the contrary, smoking exacerbates chemotherapy-induced myelosuppression through various mechanisms. Chronic inflammation and oxidative stress caused by smoking can damage bone marrow stem cells. Free radicals and toxic compounds produced by tobacco combustion induce systemic inflammatory responses, altering the bone marrow microenvironment and making it more susceptible to chemotherapeutic agents. Additionally, chemicals in tobacco, such as benzene, carbon monoxide, and nicotine, exert direct toxic effects on bone marrow stem cells by damaging DNA and interfering with cell signaling pathways, thereby inhibiting their function. Moreover, smoking suppresses immune system function, making the body more vulnerable to chemotherapy-induced bone marrow damage. Immunosuppression also increases the risk of infection, which further exacerbates myelosuppression^32^-^34^. In summary, smokers are more likely to experience severe neutropenia and thrombocytopenia during chemotherapy.

Consistent with previous studies, diabetic patients are more prone to experiencing chemotherapy-related myelosuppression^35^. The exacerbation of chemotherapy-induced myelosuppression in diabetic patients may be related to systemic chronic inflammation and metabolic disorders caused by chronic hyperglycemia. Chronic hyperglycemia in diabetic patients induces a state of low-grade systemic inflammation, primarily mediated by endotoxins, free fatty acids, and cholesterol through the activation of Toll-like receptor (TLR) and nuclear factor κB (NF-κB) pathways. This activation leads to the release of numerous pro-inflammatory cytokines that disrupt the bone marrow microenvironment, resulting in reduced production of red blood cells, white blood cells, and platelets. These inflammatory cytokines also directly induce apoptosis and dysfunction of bone marrow cells, thereby exacerbating chemotherapy-induced myelosuppression^36^-^37^.

Our predictive model indicates that overweight patients are more likely to experience chemotherapy-induced myelosuppression. Additionally, we have demonstrated the robustness and significance of this result under different statistical methods. Previous studies have shown that obese patients typically have a slower clearance rate of chemotherapeutic drugs, resulting in an extended drug elimination half-life^38^. High BMI is also usually associated with increased bone marrow fat content, which may affect the hematopoietic function of the bone marrow^36^. Contradictorily, however, many studies have shown no significant correlation between high BMI and myelosuppression^40^-^41^. Some evidence even suggests that BMI is inversely related to myelosuppression^42^. The primary reason for these conflicting findings may be related to variations in chemotherapy dosing, among other factors, and the exact mechanisms need further investigation.

Previous studies have not discussed the correlation between specific tumor locations in CRC and chemotherapy-induced myelosuppression. Our research is the first to propose this association. Our model indicates that malignancies in the rectum and sigmoid colon are more prone to chemotherapy-induced myelosuppression, which may be related to differences in treatment modalities. Rectal cancer often requires concurrent chemoradiotherapy, and the dose-volume parameters of radiotherapy (such as V20 and V25 for pelvic bone marrow) significantly affect the incidence of myelosuppression. Treatment for rectal cancer typically involves high-dose radiotherapy to the pelvic area, increasing the risk of myelosuppression. In contrast, cancers of the ascending colon, transverse colon, and descending colon generally do not require radiotherapy, with chemotherapy being the primary treatment modality. Therefore, myelosuppression in these cases is solely related to chemotherapeutic drugs.

It is evident that lung metastasis, elevated CEA, and decreased albumin levels in CRC are risk factors for chemotherapy-induced myelosuppression. We believe this is likely related to a higher tumor burden. CRC with lung metastasis has already progressed to an advanced stage, and CEA levels are proportional to the severity of CRC. A high tumor burden leads to significant albumin consumption, and such patients typically have poorer overall health, lower immune function, and reduced tolerance to chemotherapeutic drugs, making them more susceptible to myelosuppression^43^-^44^.

In this study, we chose not to include the chemotherapy regimen in the final predictive model, despite its statistical significance, as it contradicts common sense and clinical experience. Although the p-value for this factor was less than 0.05, our team's clinical experience suggests that this variable does not align with existing theories and common sense, possibly indicating a false-positive result due to data issues or chance. Additionally, previous studies have presented conflicting findings regarding the differences in myelosuppression caused by the FOLFOX, CapeOx, and FOLFIRI regimens^45^-^49^. For these reasons, we decided to exclude the chemotherapy regimen variable from the model.

### Limitations

We acknowledge the limitations of our study. First, as a retrospective investigation, our study inevitably carries selection bias, even though we applied the same inclusion and selection criteria in both the validation and training groups. Second, our findings may not be representative of all CRC patients, as the patients in this study were primarily from northern China, potentially limiting the generalizability to other populations. Furthermore, although we attempted to include all possible influencing factors, it is unavoidable that some potential factors affecting chemotherapy-induced myelosuppression were not included. Additionally, in this study, a small number of patients began interventions for myelosuppression to prevent severe cases. While this introduces some bias, it better reflects the real-world data rules. Lastly, although we performed internal validation, the robustness of our nomogram has not been verified in other groups, and its applicability to other populations of CRC patients undergoing first-line chemotherapy in different regions and countries remains uncertain. This necessitates external evaluation in a broader population. We believe it is essential to conduct further prospective studies to identify more new practical factors and validate the nomogram across multiple centers.

## Conclusions

The nomogram predictive model developed in this study, based on general information and relevant laboratory test results from colorectal cancer patients, demonstrates strong clinical decision-making capabilities. It accurately predicts the likelihood of myelosuppression after first-line chemotherapy, thereby assisting clinicians in formulating subsequent treatment plans. This approach is aimed at ensuring the smooth progression of the treatment process, reducing risks, and improving patients' quality of life.

## Figures and Tables

**Figure 3:**
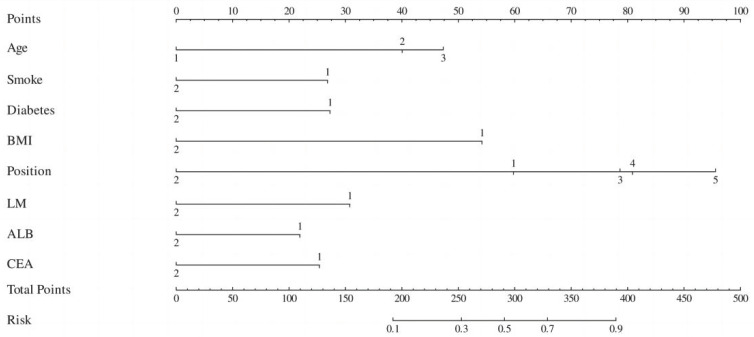
Nomogram for predicting myelosuppression induced by first-line chemotherapy in CRC. *BMI*: body mass index;* LM*: Lung metastasis; *ALB*: albumin; *CEA*: carcinoembryonic antigen. (Age: 1=<60y 2=60~74y 3=≥75y; Smoking: 1=Yes 2=Not; Diabetes: 1=Yes 2=Not: BMI: 1=<1.6 m² 2=1.6-1.8 m² 3=>1.8 m²; Location: 1=Ascending colon 2=Transverse colon 3=Descending colon 4=Sigmoid colon 5=Rectum; LM: 1=Yes 2=Not; ALB: 1=Normal 2=Abnormal; CEA: 1=Normal 2=Abnormal).

**Table 1:** Balance test between the training and validation groups.

Variables	Total (n = 855)	Validation group (n = 171)	Training group (n = 684)	Statistic	*P*	

**Age (y)**				χ²=5.27	0.072	
<60	302 (35.32)	72 (42.11)	230 (33.63)			
60~74	481 (56.26)	83 (48.54)	398 (58.19)			
≥75	72 (8.42)	16 (9.36)	56 (8.19)			
**Gender**				χ²=0.00	0.945	
Male	507 (59.30)	101 (59.06)	406 (59.36)			
Female	348 (40.70)	70 (40.94)	278 (40.64)			
**Smoking**				χ²=0.00	0.972	
Yes	306 (35.79)	61 (35.67)	245 (35.82)			
Not	549 (64.21)	110 (64.33)	439 (64.18)			
**Diabetes**				χ²=0.94	0.333	
Yes	141 (16.49)	24 (14.04)	117 (17.11)			
Not	714 (83.51)	147 (85.96)	567 (82.89)			
**Hypertension**				χ²=0.03	0.862	
Yes	345 (40.35)	68 (39.77)	277 (40.50)			
Not	510 (59.65)	103 (60.23)	407 (59.50)			
**BSA (m^2^)**				χ²=3.39	0.183	
<1.6	291 (34.04)	48 (28.07)	243 (35.53)			
1.6~1.8	309 (36.14)	67 (39.18)	242 (35.38)			
>1.8	255(29.82)	56 (32.75)	199 (29.09)			
**BMI**				χ²=2.81	0.094	
Overweight	416 (48.65)	93 (54.39)	323 (47.22)			
Not overweight	439 (51.35)	78 (45.61)	361 (52.78)			
**Tumor(T)**				χ²=1.66	0.646	
1	18 (2.11)	3 (1.75)	15 (2.19)			
2	97 (11.35)	21 (12.28)	76 (11.11)			
3	447 (52.28)	95 (55.56)	352 (51.46)			
4	293 (34.27)	52 (30.41)	241 (35.23)			
**Node(N)**				-	0.700	
0	180 (21.05)	38 (22.22)	142 (20.76)			
1	367 (42.92)	67 (39.18)	300 (43.86)			
2	303 (35.44)	65 (38.01)	238 (34.80)			
3	5 (0.58)	1 (0.58)	4 (0.58)			
**Metastasis(M)**				χ²=0.01	0.918	
0	393 (45.96)	78 (45.61)	315 (46.05)			
1	462 (54.04)	93 (54.39)	369 (53.95)			
**Tumor staging**				χ²=2.34	0.504	
I	15 (1.77)	5 (2.94)	10 (1.48)			
II	73 (8.62)	12 (7.06)	61 (9.01)			
III	296 (34.95)	58 (34.12)	238 (35.16)			
IV	463 (54.66)	95 (55.88)	368 (54.36)			
**Tumor location**				χ²=2.77	0.596	
Ascending colon	167 (19.53)	32 (18.71)	135 (19.74)			
Transverse colon	22 (2.57)	2 (1.17)	20 (2.92)			
Descending colon	28 (3.27)	4 (2.34)	24 (3.51)			
Sigmoid colon	234 (27.37)	51 (29.82)	183 (26.75)			
Rectum	404 (47.25)	82 (47.95)	322 (47.08)			
**Hepatic metastasis**				χ²=0.22	0.640	
Yes	293 (34.27)	56 (32.75)	237 (34.65)			
Not	562 (65.73)	115 (67.25)	447 (65.35)			
**Lung metastasis**				χ²=0.07	0.789	
Yes	237 (27.72)	46 (26.90)	191 (27.92)			
Not	618 (72.28)	125 (73.10)	493 (72.08)			
**Peritoneum metastasis**				χ²=0.42	0.516	
Yes	51 (5.96)	12 (7.02)	39 (5.70)			
Not	804 (94.04)	159 (92.98)	645 (94.30)			
**ALB**				χ²=0.00	0.968	
Normal	654 (76.49)	131 (76.61)	523 (76.46)			
Abnormal	201 (23.51)	40 (23.39)	161 (23.54)			
**CEA**				χ²=1.06	0.304	
Normal	455 (53.22)	85 (49.71)	370 (54.09)			
Abnormal	400 (46.78)	86 (50.29)	314 (45.91)			
**CA19-9**				χ²=0.31	0.575	
Normal	255 (29.82)	48 (28.07)	207 (30.26)			
Abnormal	600 (70.18)	123 (71.93)	477 (69.74)			
**CA125**				χ²=1.13	0.287	
Normal	89 (10.41)	14 (8.19)	75 (10.96)			
Abnormal	766 (89.59)	157 (91.81)	609 (89.04)			
**KRAS**				χ²=1.92	0.166	
Yes	140 (16.37)	34 (19.88)	106 (15.50)			
Not	715 (83.63)	137 (80.12)	578 (84.50)			
**BRAF**				χ²=0.20	0.659	
Yes	16 (1.87)	2 (1.17)	14 (2.05)			
Not	839 (98.13)	169 (98.83)	670 (97.95)			
**TP53**				χ²=0.04	0.850	
Yes	68 (7.95)	13 (7.60)	55 (8.04)			
Not	787 (92.05)	158 (92.40)	629 (91.96)			
**Myelosuppression**				χ²=0.05	0.827	
Yes	574 (67.13)	116 (67.84)	458 (66.96)			
Not	281 (32.87)	55 (32.16)	226 (33.04)			
**Chemotherapy cycle**				χ²=1.53	0.465	
First or Second	344 (40.23)	66 (38.60)	278 (40.64)			
Third or Fourth	267 (31.23)	60 (35.09)	207 (30.26)			
Fifth and Above	244 (28.54)	45 (26.32)	199 (29.09)			
**Chemotherapy regimen**				χ²=0.35	0.840	
CapeOx	639 (74.74)	126 (73.68)	513 (75.00)			
FOLFOX	93 (10.88)	18 (10.53)	75 (10.96)			
FOLFIRI	123 (14.39)	27 (15.79)	96 (14.04)			

χ²: Chi-square test, -: Fisher exact *BSA*: Body Surface Area; *BMI*: Body Mass Index; *ALB*: Albumin; *CEA*: Carcinoembryonic Antigen; *CA19-9*: Carbohydrate Antigen 19-9; *CA125*: Carbohydrate Antigen 125.

**Table 2:** Clinical characteristics of patients in both the training and validation groups.

Characteristics	Training group				Validation group		
	Myelosuppression (n=458)	Non-myelosuppression (n=226)		P -value	Myelosuppression (n=116)	Non-myelosuppression (n=55)	P -value
**Age (y)**				0.001			0.067
<60	136 (29.69%)		94 (41.59%)		43 (37.07%)	29 (52.73%)	
60~74	276 (60.26%)		122 (53.98%)		59 (50.86%)	24 (43.64%)	
≥75	46 (10.04%)		10 (4.42%)		14 (12.07%)	2 (3.64%)	
**Gender**				<.001			<.001
Male	244 (53.28%)		162 (71.68%)		58 (50.00%)	43 (78.18%)	
Female	214 (46.72%)		64 (28.32%)		58 (50.00%)	12 (21.82%)	
**Smoking**				0.001			0.134
Not	313 (68.34%)		126 (55.75%)		79 (68.10%)	31 (56.36%)	
Yes	145 (31.66%)		100 (44.25%)		37 (31.90%)	24 (43.64%)	
**Diabetes**				0.006			0.200
Not	367 (80.13%)		200 (88.50%)		97 (83.62%)	50 (90.91%)	
Yes	91 (19.87%)		26 (11.50%)		19 (16.38%)	5 (9.09%)	
**Hypertension**				0.083			0.167
Not	283 (61.79%)		124 (54.87%)		74 (63.79%)	29 (52.73%)	
Yes	175 (38.21%)		102 (45.13%)		42 (36.21%)	26 (47.27%)	
**BSA (m^2^)**				<.001			<.001
<1.6	184 (40.17%)		59 (26.11%)		39 (33.62%)	9 (16.36%)	
1.6-1.8	181 (39.52%)		61 (26.99%)		51 (43.97%)	16 (29.09%)	
>1.8	93 (20.31%)		106 (46.90%)		26 (22.41%)	30 (54.55%)	
**BMI**				<.001			<.001
Not overweight	281 (61.35%)		80 (35.40%)		52 (44.83%)	41 (74.55%)	
Overweight	177 (38.65%)		146 (64.60%)		64 (55.17%)	14 (25.45%)	
**Tumor staging**				<.001			0.032
I	4 (0.89%)		6 (2.65%)		4 (3.48%)	1 (1.82%)	
II	53 (11.75%)		8 (3.54%)		11 (9.57%)	1 (1.82%)	
III	171 (37.92%)		67 (29.65%)		44 (38.26%)	14 (25.45%)	
IV	223 (49.45%)		145 (64.16%)		56 (48.70%)	39 (70.91%)	
**Tumor location**				<.001			<.001
Ascending colon	110 (24.02%)		25 (11.06%)		31 (26.72%)	1 (1.82%)	
Transverse colon	18 (3.93%)		2 (0.88%)		1 (0.86%)	1 (1.82%)	
Descending colon	18 (3.93%)		6 (2.65%)		0 (0.00%)	4 (7.27%)	
Sigmoid colon	117 (25.55%)		66 (29.20%)		36 (31.03%)	15 (27.27%)	
Rectum	195 (42.58%)		127 (56.19%)		48 (41.38%)	34 (61.82%)	
**Hepatic metastasis**				0.046			0.730
Not	311 (67.90%)		136 (60.18%)		79 (68.10%)	36 (65.45%)	
Yes	147 (32.10%)		90 (39.82%)		37 (31.90%)	19 (34.55%)	
**Lung metastasis**				<.001			0.022
Not	355 (77.51%)		138 (61.06%)		91 (78.45%)	34 (61.82%)	
Yes	103 (22.49%)		88 (38.94%)		25 (21.55%)	21 (38.18%)	
**Peritoneum metastasis**				0.968			0.682
Not	432 (94.32%)		213 (94.25%)		109 (93.97%)	50 (90.91%)	
Yes	26 (5.68%)		13 (5.75%)		7 (6.03%)	5 (9.09%)	
**ALB**				0.001			0.047
Normal	367 (80.13%)		156 (69.03%)		94 (81.03%)	37 (67.27%)	
Abnormal	91 (19.87%)		70 (30.97%)		22 (18.97%)	18 (32.73%)	
**CEA**				<.001			0.012
Normal	222 (48.47%)		148 (65.49%)		50 (43.10%)	35 (63.64%)	
Abnormal	236 (51.53%)		78 (34.51%)		66 (56.90%)	20 (36.36%)	
**CA19-9**				0.415			0.194
Normal	134 (29.26%)		73 (32.30%)		29 (25.00%)	19 (34.55%)	
Abnormal	324 (70.74%)		153 (67.70%)		87 (75.00%)	36 (65.45%)	
**CA125**				0.643			1.000
Normal	52 (11.35%)		23 (10.18%)		9 (7.76%)	5 (9.09%)	
Abnormal	406 (88.65%)		203 (89.82%)		107 (92.24%)	50 (90.91%)	
**KRAS**				0.497			0.701
Not	384 (83.84%)		194 (85.84%)		92 (79.31%)	45 (81.82%)	
Yes	74 (16.16%)		32 (14.16%)		24 (20.69%)	10 (18.18%)	
**BRAF**				0.222			1.000
Not	446 (97.38%)		224 (99.12%)		114 (98.28%)	55 (100.00%)	
Yes	12 (2.62%)		2 (0.88%)		2 (1.72%)	0 (0.00%)	
**TP53**				0.065			0.098
Not	415 (90.61%)		214 (94.69%)		104 (89.66%)	54 (98.18%)	
Yes	43 (9.39%)		12 (5.31%)		12 (10.34%)	1 (1.82%)	
**Chemotherapy regimen**				0.111			0.002
CapeOx	174 (37.99%)		104 (46.02%)		93 (80.17%)	33 (60.00%)	
FOLFOX	142 (31.00%)		65 (28.76%)		6 (5.17%)	12 (21.82%)	
FOLFIRI	142 (31.00%)		57 (25.22%)		17 (14.66%)	10 (18.18%)	
**Chemotherapy cycle**				<.001			0.268
First or Second	371 (81.00%)		142 (62.83%)		40 (34.48%)	26 (47.27%)	
Third or Fourth	29 (6.33%)		46 (20.35%)		44 (37.93%)	16 (29.09%)	
Fifth and Above	58 (12.66%)		38 (16.81%)		32 (27.59%)	13 (23.64%)	
							

*BSA*: Body Surface Area; *BMI*: Body Mass Index; *ALB*: Albumin; *CEA*: Carcinoembryonic Antigen; *CA19-9*: Carbohydrate Antigen 19-9; *CA125*: Carbohydrate Antigen 125.

**Table 3:** Multivariate logistic regression analysis.

Intercept and variable	β	*P*	Odds ratio (95% CI)
Intercept	1.32	0.214	3.73 (0.47 ~ 29.84)
**Age (y)**			
<60			1.00 (Reference)
60~74	-0.16	0.442	0.85 (0.56 ~ 1.29)
≥75	-1.05	0.012	0.35 (0.15 ~ 0.79)
**Smoking**			
Yes			1.00 (Reference)
Not	-0.60	0.005	0.55 (0.36 ~ 0.84)
**Diabetes**			
Yes			1.00 (Reference)
Not	0.61	0.025	1.83 (1.08 ~ 3.11)
**BMI**			
Overweight			1.00 (Reference)
Not overweight	-1.21	<.001	0.30 (0.20 ~ 0.44)
**Tumor location**			
Ascending colon			1.00 (Reference)
Transverse colon	-1.33	0.121	0.26 (0.05 ~ 1.42)
Descending colon	0.42	0.477	1.52 (0.48 ~ 4.85)
Sigmoid colon	0.47	0.136	1.60 (0.86 ~ 2.96)
Rectum	0.80	0.005	2.22 (1.28 ~ 3.84)
**Tumor staging**			
I			1.00 (Reference)
II	-2.22	0.026	0.11 (0.02 ~ 0.76)
III	-1.33	0.152	0.26 (0.04 ~ 1.64)
IV	-1.42	0.133	0.24 (0.04 ~ 1.54)
**Lung metastasis**			
Yes			1.00 (Reference)
Not	-0.68	0.006	0.50 (0.31 ~ 0.82)
**ALB**			
Normal			1.00 (Reference)
Abnormal	0.49	0.032	1.63 (1.04 ~ 2.54)
**CEA**			
Normal			1.00 (Reference)
Abnormal	-0.56	0.019	0.57 (0.35 ~ 0.91)
**Chemotherapy regimen**			
CapeOx			1.00 (Reference)
FOLFOX	1.11	<.001	3.03 (1.58 ~ 5.82)
FOLFIRI	-0.13	0.650	0.88 (0.50 ~ 1.54)

β is the regression coefficient. CI: Confidence Interval *BMI*: Body Mass Index; *ALB*: Albumin; *CEA*: Carcinoembryonic antigen.

**Table 4:** Clinical characteristics of patients in both the myelosuppression and non-myelosuppression patients.

Variable	Total (n = 440)	0 (n = 220)	1 (n = 220)	Statistic	P	SMD
Age, n (%)				χ²=0.395	0.821	
1	164 (37.27)	84 (38.18)	80 (36.36)			-0.038
2	250 (56.82)	122 (55.45)	128 (58.18)			0.055
3	26 (5.91)	14 (6.36)	12 (5.45)			-0.040
Sex, n (%)				χ²=2.103	0.147	
1	306 (69.55)	160 (72.73)	146 (66.36)			-0.135
2	134 (30.45)	60 (27.27)	74 (33.64)			0.135
Smoke, n (%)				χ²=0.000	1.000	
1	180 (40.91)	90 (40.91)	90 (40.91)			0.000
2	260 (59.09)	130 (59.09)	130 (59.09)			0.000
Diabetes, n (%)				χ²=0.090	0.764	
1	50 (11.36)	26 (11.82)	24 (10.91)			-0.029
2	390 (88.64)	194 (88.18)	196 (89.09)			0.029
Hypertension, n (%)				χ²=0.758	0.384	
1	183 (41.59)	96 (43.64)	87 (39.55)			-0.084
2	257 (58.41)	124 (56.36)	133 (60.45)			0.084
BSA, n (%)				χ²=1.692	0.429	
1	117 (26.59)	54 (24.55)	63 (28.64)			0.090
2	152 (34.55)	82 (37.27)	70 (31.82)			-0.117
3	171 (38.86)	84 (38.18)	87 (39.55)			0.028
BMI, n (%)				χ²=0.231	0.631	
1	249 (56.59)	122 (55.45)	127 (57.73)			0.046
2	191 (43.41)	98 (44.55)	93 (42.27)			-0.046
Tumor(T), n (%)				-	0.314	
1	1 (0.23)	0 (0.00)	1 (0.45)			0.068
2	51 (11.59)	30 (13.64)	21 (9.55)			-0.139
3	234 (53.18)	111 (50.45)	123 (55.91)			0.110
4	154 (35)	79 (35.91)	75 (34.09)			-0.038
Node(N), n (%)				-	0.374	
0	79 (17.95)	35 (15.91)	44 (20.00)			0.102
1	212 (48.18)	110 (50.00)	102 (46.36)			-0.073
2	147 (33.41)	75 (34.09)	72 (32.73)			-0.029
3	2 (0.45)	0 (0.00)	2 (0.91)			0.096
Metastasis(M), n (%)				χ²=0.091	0.763	
0	151 (34.32)	74 (33.64)	77 (35.00)			0.029
1	289 (65.68)	146 (66.36)	143 (65.00)			-0.029
Staging, n (%)				-	0.382	
1	4 (0.91)	2 (0.91)	2 (0.91)			0.000
2	12 (2.73)	3 (1.36)	9 (4.09)			0.138
3	131 (29.77)	67 (30.45)	64 (29.09)			-0.030
4	293 (66.59)	148 (67.27)	145 (65.91)			-0.029
Position, n (%)				χ²=0.756	0.944	
1	47 (10.68)	22 (10.00)	25 (11.36)			0.043
2	8 (1.82)	5 (2.27)	3 (1.36)			-0.078
3	19 (4.32)	9 (4.09)	10 (4.55)			0.022
4	137 (31.14)	69 (31.36)	68 (30.91)			-0.010
5	229 (52.05)	115 (52.27)	114 (51.82)			-0.009
HM, n (%)				χ²=0.755	0.385	
1	185 (42.05)	97 (44.09)	88 (40.00)			-0.084
2	255 (57.95)	123 (55.91)	132 (60.00)			0.084
LM, n (%)				χ²=0.254	0.614	
1	149 (33.86)	72 (32.73)	77 (35.00)			0.048
2	291 (66.14)	148 (67.27)	143 (65.00)			-0.048
PM, n (%)				χ²=0.164	0.686	
1	26 (5.91)	14 (6.36)	12 (5.45)			-0.040
2	414 (94.09)	206 (93.64)	208 (94.55)			0.040
ALB, n (%)				χ²=0.106	0.745	
1	325 (73.86)	164 (74.55)	161 (73.18)			-0.031
2	115 (26.14)	56 (25.45)	59 (26.82)			0.031
CEA, n (%)				χ²=0.356	0.551	
1	282 (64.09)	138 (62.73)	144 (65.45)			0.057
2	158 (35.91)	82 (37.27)	76 (34.55)			-0.057
CA199, n (%)				χ²=0.010	0.920	
1	151 (34.32)	75 (34.09)	76 (34.55)			0.010
2	289 (65.68)	145 (65.91)	144 (65.45)			-0.010
CA125, n (%)				χ²=0.084	0.771	
1	54 (12.27)	28 (12.73)	26 (11.82)			-0.028
2	386 (87.73)	192 (87.27)	194 (88.18)			0.028
KRAS, n (%)				χ²=1.087	0.297	
1	51 (11.59)	22 (10.00)	29 (13.18)			0.094
2	389 (88.41)	198 (90.00)	191 (86.82)			-0.094
BRAF, n (%)				χ²=0.000	1.000	
1	3 (0.68)	2 (0.91)	1 (0.45)			-0.068
2	437 (99.32)	218 (99.09)	219 (99.55)			0.068
TP53, n (%)				χ²=0.927	0.336	
1	18 (4.09)	7 (3.18)	11 (5.00)			0.083
2	422 (95.91)	213 (96.82)	209 (95.00)			-0.083
CF, n (%)				χ²=0.842	0.656	
1	182 (41.36)	88 (40.00)	94 (42.73)			0.055
2	143 (32.5)	76 (34.55)	67 (30.45)			-0.089
3	115 (26.14)	56 (25.45)	59 (26.82)			0.031
CR, n (%)				χ²=1.822	0.402	
1	307 (69.77)	160 (72.73)	147 (66.82)			-0.125
2	73 (16.59)	33 (15.00)	40 (18.18)			0.082
3	60 (13.64)	27 (12.27)	33 (15.00)			0.076

*BSA*: Body Surface Area; *BMI*: Body Mass Index; *ALB*: Albumin; *CEA*: Carcinoembryonic Antigen; *CA19-9*: Carbohydrate Antigen 19-9;* CA125*: Carbohydrate Antigen 125.

**Table 5:** Multivariate logistic regression analysis after PSM

Variables	β	P	OR (95%CI)
Intercept	-1.25	0.549	0.29 (0.00 ~ 17.24)
Age			
1			1.00 (Reference)
2	-0.00	0.990	1.00 (0.62 ~ 1.60)
3	-0.61	0.200	0.55 (0.22 ~ 1.38)
Sex			
1			1.00 (Reference)
2	-0.26	0.485	0.77 (0.37 ~ 1.61)
Smoke			
1			1.00 (Reference)
2	-0.18	0.548	0.84 (0.47 ~ 1.50)
Diabetes			
1			1.00 (Reference)
2	0.39	0.219	1.48 (0.79 ~ 2.75)
Hypertension			
1			1.00 (Reference)
2	-0.00	0.993	1.00 (0.63 ~ 1.59)
BSA			
1			1.00 (Reference)
2	-0.21	0.532	0.81 (0.41 ~ 1.58)
3	-0.03	0.942	0.97 (0.41 ~ 2.29)
BMI			
1			1.00 (Reference)
2	-0.71	0.008	0.49 (0.29 ~ 0.83)
Tumor(T)			
1			1.00 (Reference)
2	1.39	0.260	4.02 (0.36 ~ 45.48)
3	1.50	0.215	4.50 (0.42 ~ 48.34)
4	1.37	0.259	3.93 (0.36 ~ 42.32)
Node(N)			
0			1.00 (Reference)
1	-0.02	0.952	0.98 (0.48 ~ 1.99)
2	-0.28	0.473	0.75 (0.35 ~ 1.64)
Metastasis(M)			
0			1.00 (Reference)
1	-0.72	0.562	0.49 (0.04 ~ 5.52)
Staging			
1			1.00 (Reference)
2	-1.51	0.164	0.22 (0.03 ~ 1.85)
3	-0.95	0.366	0.39 (0.05 ~ 3.03)
4	-0.17	0.917	0.84 (0.03 ~ 20.45)
Position			
1			1.00 (Reference)
2	-0.73	0.450	0.48 (0.07 ~ 3.20)
3	0.12	0.846	1.13 (0.34 ~ 3.78)
4	0.19	0.629	1.21 (0.56 ~ 2.63)
5	0.44	0.197	1.55 (0.80 ~ 3.01)
HM			
1			1.00 (Reference)
2	0.07	0.823	1.07 (0.59 ~ 1.93)
LM			
1			1.00 (Reference)
2	-0.37	0.218	0.69 (0.39 ~ 1.24)
PM			
1			1.00 (Reference)
2	-0.10	0.837	0.91 (0.36 ~ 2.31)
ALB			
1			1.00 (Reference)
2	0.28	0.253	1.33 (0.82 ~ 2.17)
CEA			
1			1.00 (Reference)
2	-0.47	0.104	0.63 (0.36 ~ 1.10)
CA199			
1			1.00 (Reference)
2	0.05	0.858	1.05 (0.62 ~ 1.78)
CA125			
1			1.00 (Reference)
2	0.33	0.345	1.39 (0.70 ~ 2.75)
KRAS			
1			1.00 (Reference)
2	0.03	0.933	1.03 (0.50 ~ 2.12)
BRAF			
1			1.00 (Reference)
2	0.15	0.903	1.16 (0.11 ~ 11.99)
TP53			
1			1.00 (Reference)
2	0.28	0.608	1.33 (0.45 ~ 3.89)
CF			
1			1.00 (Reference)
2	-0.13	0.581	0.87 (0.54 ~ 1.41)
3	-0.28	0.270	0.75 (0.45 ~ 1.25)
CR			
1			1.00 (Reference)
2	0.56	0.140	1.76 (0.83 ~ 3.71)
3	-0.26	0.454	0.77 (0.39 ~ 1.53)

OR: Odds Ratio, CI: Confidence Interval *BSA*: Body Surface Area; *BMI*: Body Mass Index; *ALB*: Albumin; *CEA*: Carcinoembryonic Antigen; *CA19-9*: Carbohydrate Antigen 19-9; *CA125*: Carbohydrate Antigen 125.

## Abbreviations

CRC: Colorectal cancer
BSA: body surface area
BMI: body mass index
T: Tumor
N: Node
M: Metastasis
ALB: albumin
CA125: carbohydrate antigen 125
CA19-9: carbohydrate antigen 19-9
CEA: carcinoembryonic antigen
AJCC: the American Joint Committee on Cancer
LASSO: the Least Absolute Shrinkage and Selection Operator
AIC: Akaike Information Criterion
ROC: the receiver operating characteristic
DCA: decision curve analysis
AUC: the area under the ROC curve
DNA: deoxyribonucleic acid
TLR: the activation of Toll-like receptor
NF-κB: nuclear factor κB
